# CD8^+^ T cells inhibit metastasis and CXCL4 regulates its function

**DOI:** 10.1038/s41416-021-01338-5

**Published:** 2021-04-01

**Authors:** Robiya Joseph, Rama Soundararajan, Suhas Vasaikar, Fei Yang, Kendra L. Allton, Lin Tian, Petra den Hollander, Sevinj Isgandarova, Monika Haemmerle, Barbara Mino, Tieling Zhou, Crystal Shin, Melisa Martinez-Paniagua, Aysegul A. Sahin, Jaime Rodriguez-Canales, Juri Gelovani, Jeffrey T. Chang, Ghanashyam Acharya, Anil K. Sood, Ignacio I. Wistuba, Don L. Gibbons, Luisa M. Solis, Michelle C. Barton, Navin Varadarajan, Jeffrey M. Rosen, Xiang H. Zhang, Sendurai A. Mani

**Affiliations:** 1 Department of Translational Molecular Pathology, The University of Texas MD Anderson Cancer Center, Houston, TX USA; 2 Department of Epigenetics and Molecular Carcinogenesis, The University of Texas MD Anderson Cancer Center, Houston, TX USA; 3 Department of Molecular and Cellular Biology, Baylor College of Medicine, Houston, TX USA; 4 Institute of Biosciences and Technology, Texas A&M Health Science Center, Houston, TX USA; 5 Department of Gynecologic Oncology and Reproductive Medicine and Cancer Biology, The University of Texas MD Anderson Cancer Center, Houston, TX USA; 6 Department of Surgery, Baylor College of Medicine, Houston, TX USA; 7 Department of Chemical and Biomolecular Engineering, University of Houston, Houston, TX USA; 8 Department of Pathology, Division of Pathology/Lab Medicine, The University of Texas MD Anderson Cancer Center, Houston, TX USA; 9 Department of Experimental Diagnostic Imaging, Wayne State University, Detroit, MI USA; 10 Department of Integrative Biology and Pharmacology, UT Health Sciences Center at Houston, Houston, TX USA; 11 Department of Thoracic Head and Neck Medical Oncology, Division of Cancer Medicine Division, The University of Texas MD Anderson Cancer Center, Houston, TX USA; 12 Present Address: Sloan Kettering Institute, New York, NY USA; 13 Present Address: University Clinic Halle, Institute of Pathology, Halle, Germany; 14 Present Address: AstraZeneca, Gaithersburg, MD USA

**Keywords:** Tumour immunology, Metastasis

## Abstract

**Background:**

The mechanism by which immune cells regulate metastasis is unclear. Understanding the role of immune cells in metastasis will guide the development of treatments improving patient survival.

**Methods:**

We used syngeneic orthotopic mouse tumour models (wild-type, NOD/scid and Nude), employed knockout (*CD8* and *CD4*) models and administered CXCL4. Tumours and lungs were analysed for cancer cells by bioluminescence, and circulating tumour cells were isolated from blood. Immunohistochemistry on the mouse tumours was performed to confirm cell type, and on a tissue microarray with 180 TNBCs for human relevance. TCGA data from over 10,000 patients were analysed as well.

**Results:**

We reveal that intratumoral immune infiltration differs between metastatic and non-metastatic tumours. The non-metastatic tumours harbour high levels of CD8^+^ T cells and low levels of platelets, which is reverse in metastatic tumours. During tumour progression, platelets and CXCL4 induce differentiation of monocytes into myeloid-derived suppressor cells (MDSCs), which inhibit CD8^+^ T-cell function. TCGA pan-cancer data confirmed that CD8^low^Platelet^high^ patients have a significantly lower survival probability compared to CD8^high^Platelet^low^.

**Conclusions:**

CD8^+^ T cells inhibit metastasis. When the balance between CD8^+^ T cells and platelets is disrupted, platelets produce CXCL4, which induces MDSCs thereby inhibiting the CD8^+^ T-cell function.

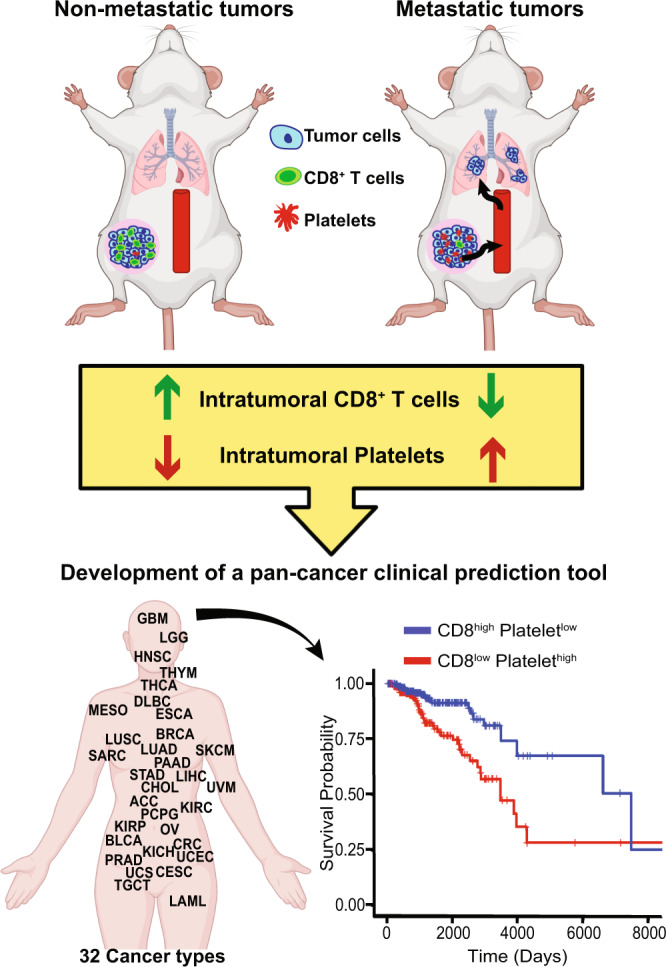

## Background

Metastasis causes the majority of cancer-related deaths.^1^ During metastasis, tumour cells invade surrounding tissues, enter the vasculature, survive the sheer force of blood flow, extravasate at a distant site and develop into metastatic tumours.^2–6^ The dynamic interplay between tumour cells and immune cells within the tumour microenvironment (TME) is a critical determinant of whether or not a tumour will metastasise.^7–16^ Within the primary tumour, immune cells play a critical and dynamic role in regulating the primary tumour growth and dissemination of tumour cells into the circulation.^11,15,17^ However, the mechanism by which the immune cells regulate metastasis, and in particular, the dissemination of tumour cells from the primary tumour site into circulation, is not well-understood. Understanding the role of immune cells in metastasis may help guide the development of novel treatment options to improve the survival of cancer patients with metastatic disease.

In the past decade, immunotherapies have had a major impact on clinical oncology.^18,19^ Different approaches to immunotherapy are under development including monoclonal antibodies, cytokines and adoptive T-cell therapy. This field is constantly evolving based on the preclinical data and clinical requirements. The effectiveness of immunotherapy depends on the ability of checkpoint modulators to unleash pre-existing immunity, specifically T-cell effector functionality.^20,21^ T cells from the lymphoid compartment have been shown to regulate metastasis.^22–24^ Inhibiting the checkpoint modulators is an important tool in cancer immunotherapy and well-established checkpoint molecules include programmed cell death 1 (PD-1), programmed cell death 1 ligand 1 (PD-L1), cytotoxic T lymphocyte-associated protein 4 (CTLA-4), T-cell immunoglobulin and mucin domain 3 (TIM-3) and lymphocyte activation gene 3 (LAG3). T cells upregulate the expression of these molecules in a classical adaptive response within the TME. Among the T cells, CD8^+^ T cells significantly contribute to the effector functions of adaptive immunity.^25^ The contribution of CD8^+^ T cells to metastasis, which is currently not well established, is the main core of our study.

From the myeloid lineage, platelets that originate from the cytoplasm of megakaryocytes are major cellular mediators of haemostasis and thrombosis and are important modulators of tumour progression and metastasis.^26^ When platelets are activated, they produce several factors, including CXCL4, also known as chemokine C–X–C motif ligand 4 or platelet factor 4 (PF4).^27^ The contribution of CXCL4 to metastasis has not been investigated. Even though a lot is known about the role of platelets in metastasis,^28,29^ how they impact the immune cells and in particular, the CD8^+^ T cells during metastasis is not clear.

In this paper, we demonstrate how the immune system influences metastasis. We show that CD8^+^ T cells are the critical inhibitors of metastasis. We discovered that CXCL4, secreted by platelets^30,31^, promotes the differentiation of monocytes into MDSCs and that these MDSCs in turn inhibit CD8^+^ T cells. By analysing Cancer Genome Atlas (TCGA) data, we found a strong inverse correlation between CD8^+^ T cells and platelets, and that patients with low CD8^+^ T cells and high platelets are more likely to develop metastases. Thus, this study for the first time demonstrates the inhibitory role of CD8^+^ T cells in the interplay among platelets, MDSCs and CD8^+^ T cells. This inhibitory effect serves as a checkpoint for the entry of tumour cells into circulation to initiate the metastatic process.

## Methods

### Mice

Wild-type female BALB/cJ mice (stock no. 0000651) and immunocompromised female NOD.CB17-Prkdc scid/J mice (stock no. 001303) were purchased from Jackson Laboratory. BALB/c nude mice (CAnN.Cg-Foxn1nu/Crl, strain code 194) were purchased from Charles River Laboratories. *CD8*-knockout and *CD4*-knockout BALB/cJ mice were kindly provided by Dr. Xiang Zhang, Baylor College of Medicine. Animal experiments were performed in accordance with the guidelines put forth, verified and approved by the Institutional Animal Care and Use Committee of the University of Texas MD Anderson Cancer Center.

### Cell lines

Isogenic 67NR and 4T1 cell lines were obtained from Karmanos Cancer Institute. Cells were shown to be free of mycoplasma. Cells were cultured in DMEM-F/12 with 10% foetal bovine serum (FBS) and 5% penicillin/streptomycin. Experiments were performed on cells within 2 weeks of thawing. 67NR and 4T1 cells were engineered to stably express firefly luciferase and RFP by stable transfection with the pMIR-luciferase-RFP plasmid.

### Spontaneous breast cancer metastasis model

The abdominal fur was trimmed, and the abdomen was wiped with 70% EtOH to expose the nipples. 67NR or 4T1 cells (1 × 10^4^ cells per mouse) were injected into the fourth pair of mammary fat pads. The mice were subjected to whole-body bioluminescence imaging weekly. The experiment was terminated at around 4 weeks when the primary tumour volume exceeded 2 cm^3^. The mice were anaesthetised with isoflurane followed by euthanasia by cervical dislocation, and the primary tumour, lungs and blood were harvested. The tumour volume was calculated using the formula *L* × (*W* × *W*)/2, where *L* is the length and *W* is the width of the tumour, as previously described.^32^ Primary tumours and lungs were processed for bioluminescence imaging and immunohistochemistry. Blood samples were analysed for CTCs.

### Experimental metastasis

67NR or 4T1 cells (1 × 10^4^ cells per mouse) were injected into the tail vein. The mice were subjected to whole-body bioluminescence imaging weekly. The experiment was terminated between 3 and 4 weeks post tumour cell injection based on the condition of the mice. Upon termination, the lungs and blood were collected.

### CD8^+^ T-cell depletion

To deplete the BALB/c wild type mice of CD8^+^ T cells, we performed i.p. injections of 250 µg anti-mouse CD8a (BioXCell, Clone 1A8, BE0004-1) at days 0, 3, 10 and 17 after the injection of 67NR cells to the fourth mammary fat pad. A rat IgG2a isotype control (BioXCell, Clone 2A3, BE0089) was administered in the same way to animals of the control group. The mice were subjected to whole-body bioluminescence imaging weekly. The experiment was terminated between 3 and 4 weeks based on the condition of the mice. Upon termination, the lungs and blood were collected.

### Treatment of mice with CXCL4

In order to study the effect of CXCL4 on metastasis of 67NR in an immunocompetent mouse model, 1 × 10^4^ of 67NR cells were injected into the fourth pair of mammary fat pads of BALB/cJ mice. Recombinant CXCL4 (BioLegend, catalogue number 590202) was injected (20 µg/mouse) intraperitoneally (i.p.) on the same day as tumour cell injection, day 1, and on every alternate day until termination at day 21. The control group was treated with phosphate-buffered saline, pH 7.4 (PBS). Upon termination of the experiment, the primary tumours, lungs and blood were collected.

### Tissue microarray

Formalin-fixed and paraffin-embedded samples of surgically resected breast cancer specimens were obtained from Breast Tumor Bank at MD Anderson Cancer Center.^33^ Tumour tissue specimens obtained from 180 breast cancers during the period from 2001 to 2013 and were histologically examined and classified using the World Health Organisation classification of breast tumours. Tissue microarrays were constructed with three 1-mm-diameter cores per tumour. Clinical and pathologic information, including demographic, pathologic TNM staging, overall survival and time of recurrence are available for each patient.

### Immunohistochemistry staining and image analysis

Immunocytochemistry was performed using an automated BOND-MAX staining system (Leica Microsystems) with antibodies against human CD8 (Thermo Fisher Scientific, Clone C8/144B, MS-457-S), mouse CD8 (Cell Signaling Technology, D4W2Z, 98941S), human CD61 (Cell Marque, Clone 2F2, 161M), and mouse CD61 (Cell Signaling Technology, D7X3P, 13166T). After scanning using a ScanScope Aperio AT Turbo slide scanner (Leica Microsystems), the slides were visualised using the ImageScope software (Leica Microsystems) and analysed using the Aperio Image Analysis Software (Leica Microsystems), as previously described.^34^ For whole tissues from mouse, five randomly selected square areas (1 mm^2^ each) in the tumour were evaluated. The average total number of cells positive for each marker in the five areas were expressed in density per mm^2^. In the tissue microarrays, the average of the total number of positive cells in the three cores from the same patient was determined in density per mm^2^.

### Cytokine analysis

Tumours were harvested, and lysates were processed and analysed using the mouse cytokine array (RayBiotech, catalogue number AAM-CYT-3). The signal intensities (in arbitrary units) were normalised to the signal from positive control spots using Image J software.^35^

### Antibodies, flow cytometry and FACS sorting

The following fluorochrome-conjugated antibodies from BioLegend were used: FITC-labelled rat IgG2b (catalogue number 400633), FITC-labelled anti-mouse Gr-1 (catalogue number 108406), brilliant violet 421-labelled rat IgG2b (catalogue number 400639), brilliant violet 421-labelled anti-mouse/human CD11b (catalogue number 101235), APC/Cy7-labelled rat IgG2a (catalogue number 400523), APC/Cy7-labelled anti-mouse CD8a (catalogue number 100713), PE-labelled rat IgG2b (catalogue number 400607), PE-labelled anti-mouse/human CD11b (catalogue number 101207), Alexa Fluor 488-labelled anti-mouse CD3 (catalogue number 100212), Alexa Fluor 488-labelled anti-mouse CD4 (catalogue number 100425), Alexa Fluor 488-labelled anti-mouse CD8a (catalogue number 100723) and PE-labelled anti-mouse CD45 (catalogue number 103106). For viability analysis, NucRed Dead 647 ReadyProbes Reagent (ThermoFisher Scientific, catalogue number R37113) was used. Cells (5 × 10^5^ per sample) were resuspended in 100 µl of FACS buffer (PBS with 4% FBS), and 1 µg/ml of the desired antibody was added. For MDSC analysis, prior to the addition of the antibody of interest, an Fc block (rat anti-mouse CD16/CD32, BD Pharmingen, catalogue number 553142; 1:200) was added to the cells, and cells were incubated at 4 °C for 30 min. The cells were incubated for 30 min in the dark at room temperature and then washed three times with FACS buffer and centrifuged at 400 × *g* for 5 min. The cells were then resuspended in 300–500 µl of FACS buffer and analysed on an Lsr2 analyser or sorted on BD FacsFusion machine.

### Mass cytometry analyses

Tumours were harvested and digested in warm digestion buffer containing 5 mg of hyaluronidase and 30 mg of collagenase type 1A (both from Sigma-Aldrich) in 10 ml of DMEM/F12 with shaking at 37 °C for 30 min. The samples were washed and fixed in 2% paraformaldehyde. Each sample was barcoded utilising cisplatin metals (a combination of ^194^Pt, ^195^Pt and ^196^Pt, Fluidigm, provided by Dr. Michelle Barton’s lab), as previously described.^36^ Staining was completed as previously described.^37^ Briefly, the samples were stained with cell-surface antibodies for 1 h at room temperature and permeabilised with chilled methanol overnight. The intracellular antibody staining was then performed for 1 h at room temperature followed by incubation with the Ir-intercalator (^191^Ir/^193^Ir) stain for 15 min. The antibodies utilised for cell-surface staining were the following: anti-CD45 (isotope ^89^Y, DVS-Fluidigm, catalogue number 3089005B), anti-CD4 (isotope ^115^In, BioLegend, catalogue number 100506), anti-TCR Va2 (isotope ^139^La, BioLegend, catalogue number 127802), anti-CD39 (isotope ^142^Nd, DVS-Fluidigm, catalogue number 3142005B), anti-CD183 (isotope ^143^Nd, BioLegend, catalogue number 126502), anti-CD115 (isotope ^144^Nd, DVS-Fluidigm, catalogue number 3144012B), anti-CD8 (isotope ^146^Nd, BioLegend, catalogue number 100702), anti-CD103 (isotype ^147^Sm, BioLegend, catalogue number 121401), anti-CD27 (isotope ^148^Nd, BioLegend, catalogue number 124202), anti-CD19 (isotope ^149^Sm, BioLegend, catalogue number 115502), anti-Ly-6C (isotope ^150^Nd, DVS-Fluidigm, catalogue number 3150010B), anti-CD123 (isotope ^151^Eu, BioLegend, catalogue number 106002), anti-CD3 (isotope ^152^Sm, BioLegend, catalogue number 100302), anti-CD274 (isotope ^153^Eu, DVS-Fluidigm, catalogue number 3153016B), anti-CCR7 (isotope ^155^Gd, eBioscience, catalogue number 16-1971-85), anti-CD69 (isotope ^156^Gd, BioLegend, catalogue number 104533), anti-F4/80 (isotope 159Tb, BioLegend, catalogue number 123102), anti-TCRgd (isotope ^160^Gd, eBioscience, catalogue number 14-5711-82), anti-TIM-3 (isotype ^162^Dy, DVS-Fluidigm, catalogue number 3162029B), anti-CD223 (isotype ^163^Dy, BioLegend, catalogue number 125202), anti-CD62L (isotope ^164^Dy, DVS-Fluidigm, catalogue number 3164003B), anti-CD31 (isotope ^165^Ho, DVS-Fluidigm, catalogue number 3165013B), anti-Arginase-1 (isotope ^166^Er, BioLegend, catalogue number 678802), anti-CD14 (isotype ^169^Tm, BioLegend, catalogue number 123302), anti-NK1.1 (isotype 170Er, BioLegend, catalogue number 108702), anti-CD279 (isotope ^171^Yb, BioLegend, catalogue number 135202), anti-CD11b (isotope ^172^Yb, DVS-Fluidigm, catalogue number 3172012B), anti-I-A/I-E (isotope 174Yb, DVS-Fluidigm, catalogue number 3174003B), anti-Ly6-G (isotope ^175^Lu, BioLegend, catalogue number 108402), anti-CD278 (isotope ^176^Yb, DVS-Fluidigm, catalogue number 3176014B) and anti-CD11c (isotope ^209^Bi, DVS-Fluidigm, catalogue number 3209005B). The antibodies used for intracellular staining were the following: anti-iNOS (isotope ^141^Pr, Abcam, catalogue number ab239990), anti-GATA3 (isotope ^145^Nd, eBioscience, catalogue number 14-9966-82), anti-IFNg (isotope ^154^Sm, DVS-Fluidigm, catalogue number 3165003B), anti-Foxp3 (isotope ^158^Gd, DVS-Fluidigm, catalogue number 3158003A), anti-T bet (isotope 161Dy, DVS-Fluidigm, catalogue number 3161014B), anti-IL-6 (isotope ^167^Er, DVS-Fluidigm, catalogue number 3167003B), anti-Ki67 (isotope ^168^Er, BD Biosciences, catalogue number 556003) and anti-Granzyme B (isotope ^173^Yb, DVS-Fluidigm, catalogue number 3173006B). The samples were analysed on the CyTOF Helios-081 mass cytometer. Data processing, gating and visualisation were performed using FlowJo v10.6.1 and Cytobank (Beckman).

### ELISA

The CXCL4 ELISA (R&D Systems, MCX400) was performed using lysates of cultured cells and tumour tissue as described by the manufacturer and was read on a microplate reader at 450 nm. The graph with the concentration of CXCL4 was generated using GraphPad Prism.

### Generation of MDSCs from the bone marrow

Bone marrow cells isolated from wild-type BALB/c mice were suspended at 2.5 × 10^5^ cells in complete RPMI media with GM-CSF (40 ng/ml; R&D Systems, catalogue number 415-ML-010), CXCL4 (10 µg/ml; BioLegend, catalogue number 590202) or platelets (2 × 10^5^) and were cultured at 37 °C for 4 days. The cells were then harvested and stained using anti-CD11b and anti-Gr-1 antibodies, markers of MDSCs and analysed on an Lsr2 analyser.

### CFSE T-cell proliferation assay

CD8^+^ T cells were quantified by isolating T cells from the spleens of wild-type BALB/c mice and stained with 10 μM of cell proliferation dye eFluor 450 and incubated at 37 °C for 4 days. The cells were then harvested and stained for CD8^+^ T cells using CD8 antibody and FACS analysed for CD8^+^ and eFluor 450.

### MDSC functional assay

T cells were isolated from the spleens of wildtype BALB/c mice, stained with eFluor 450 and activated by culturing in the presence of 3 mg/ml of anti-CD3 antibody (BioLegend, catalogue number 100302) and 1 mg/ml of anti-CD28 antibody (BioLegend, catalogue number 102102) as previously described.^38^ Cells were then incubated with MDSCs for 4 days at 37 °C. CD8^+^ T-cell proliferation was then analysed.

### TCGA patient data

We downloaded the TCGA RNA-seq normalised count data and clinical characteristics for 32 cancer types, including breast (http://gdac.broadinstitute.org/, January 2016 version). Data on primary solid tumours were used for analysis.

### Cell abundance determination

CD8^+^ T cell and platelet abundances for each patient were inferred using the ssGSEA method implemented in GSVA method^39^ using immune markers gene set previously published.^40^ The platelet signature is the combination of expression of *CD36*, *ITGA2B*, *GP9*, *SELP*, *ITGB3*, *GP1BA*, *ADCYAP1R1*, *CD63*, *SELL* and *SELPLG.*^41^

### Survival analysis

Survival for each patient in each TCGA cancer cohort was calculated as the time from date of diagnosis to death or last contact. The Kaplan–Meier method and the log-rank test were used to analyse differences in survival time based on the expression of *CD8A* (CD8^+^ T cells) and *ITGB3* (platelets). Normalised RNA expression of each immune marker was categorised into low and high expression based on the median. The p values were determined using a log-rank test. Univariate Cox proportional hazards models were fitted to calculate the hazard ratios using coxph function in the Survival package (version 2.44).

### Statistical analyses

Correlation and survival analyses were performed using cor.test and survival function in R (Bioconductor, version 3.4). The *t* tests were performed using GraphPad Prism. The correlations between cell abundances were determined using Spearman’s correlation function implemented in R (Bioconductor, version 3.4). *P* values < 0.05 were considered to be statistically significant.

## Results

### Direct inoculation of non-metastatic tumour cells into the circulation makes them metastatic

**Fig. 1:**
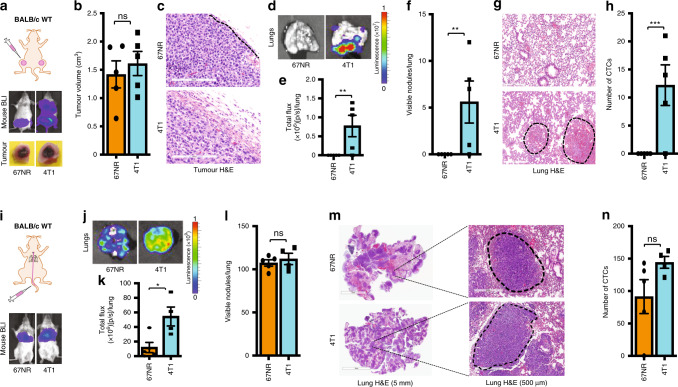
Direct inoculation of non-metastatic tumour cells into the circulation makes them metastatic. In wild-type BALB/c mice, 67NR breast cancer cells are unable to metastasise to the lung from the mammary fat pad, while 4T1 completes the entire cascade (**a**–**h**). **a** A cartoon showing the mammary fat pad injection (top). Representative whole-body bioluminescence image of wild-type BALB/c mice harbouring 67NR or 4T1 tumours at the orthotopic mammary fat-pad (middle) and excised tumours (bottom) (*n* = 5). **b** The mean tumour volume of the tumours excised from these mice (*n* = 5). **c** Representative H&E staining of 67NR and 4T1 tumours from BALB/c mice (*n* = 5). Scale bar: 200 µm. **d** Representative bioluminescence images of lungs excised from BALB/c mice harbouring 67NR or 4T1 tumours at the orthotopic mammary fat-pad (top) and **e** the mean total flux from these lungs (bottom) (*n* = 5). **f** The number of visible lung nodules in 67NR and 4T1 tumour-bearing BALB/c mice (*n* = 5). **g** Representative haematoxylin and eosin (H&E)-stained lung tissue from 67NR and 4T1 tumour-bearing BALB/c mice. Scale bar: 300 µm. **h** The number of CTCs isolated from BALB/c mice harbouring 67NR and 4T1 tumours at the mammary fat pad (*n* = 5). Non-metastatic 67NR cancer cells metastasise to the lung similar to metastatic 4T1 cells when introduced via tail vein in wildtype BALB/c mice (**i**–**n**). **i** A cartoon showing the tail vein injection (top). Representative whole-body bioluminescence images of BALB/c mice injected with 67NR and 4T1 via the tail vein (bottom). **j** Representative bioluminescence images of lungs excised from BALB/c injected with 67NR and 4T1 via tail vein (top). **k** The mean total flux from these lungs (bottom) (*n* = 5 per group). **l** The number of visible lung nodules of BALB/c mice injected with 67NR and 4T1 via tail vein (*n* = 5). **m** H&E staining of representative lungs from BALB/c mice injected with 67NR and 4T1 via the tail vein. Scale bar: 5 mm and 500 µm. **n** The number of CTCs isolated from BALB/c mice injected with 67NR and 4T1 via tail vein (*n* = 5). Quantitative data are mean ± SEM. Statistical analyses were performed by unpaired two-tailed Student’s *t* test; ns indicates not significant, ^∗^*P* ≤ 0.05, ^∗∗^*P* ≤ 0.01, ^∗∗∗^*P* ≤ 0.001.

The entry of tumour cells into the circulation is a rate-limiting step in metastasis,^11^ and this entry is regulated by the local tumour microenvironment (TME). To examine the role of TME in regulating invasion and metastasis of tumour cells, we utilised syngeneic mammary tumour cell models (67NR and 4T1) with equivalent primary tumour-forming ability at the orthotopic site (the mammary gland) in wild-type BALB/c mice, but with different metastatic potentials.^42,43^ As reported previously,^42^ these two cell lines grew with equal efficiency in vitro (Supplementary Fig. S1a), formed the same number of colonies in soft agar (Supplementary Fig. S1b), and the same number of acini (Supplementary Fig. S1c), and formed primary tumours with equal efficiency in the mammary gland (Fig. 1a, b). In addition, staining of 67NR and 4T1 primary tumours for CD31 revealed that there were no significant differences in the neovasculature (Supplementary Fig. S1d, e). We also stained the 67NR and 4T1 tumours for Ki67 and observed no differences in this proliferation marker between these two tumours (Supplementary Fig. S1f, g). Staining of the primary tumours with haematoxylin and eosin (H&E) revealed that 67NR primary tumours had clear demarked boundaries, whereas the 4T1 tumour cells invaded the muscle wall (Fig. 1c). Furthermore, 67NR cells did not metastasise to any organ, whereas the 4T1 cells metastasised to the lungs as evidenced by the luminescence signal in the lungs removed from tumour-bearing mice (Fig. 1d, e). There were no visible lung nodules in the 67NR tumour-bearing mice, whereas 4T1 tumour-bearing mice had six or more nodules per lung (Fig. 1f). H&E staining of the lungs revealed similar results (Fig. 1g). In addition, we did not observe any circulating tumour cells (CTCs) in the 67NR tumour-bearing mice, but more than ten CTCs per 0.5 ml of blood were detected in 4T1 tumour-bearing mice (Fig. 1h). While these findings reconfirmed the original observation made by Miller,^42^ it also raised an important question, i.e., why the 67NR cells are non-metastatic, even though they both are isogenic cell lines.

To metastasise, tumour cells must survive in the circulation in the absence of extracellular matrix components. To evaluate the abilities of 4T1 and 67NR cells to survive in suspension, we cultured the cells in appropriate culture media in non-adherent cell-culture plates. Surprisingly, the 67NR cells were able to survive in suspension, as did the 4T1 cells (Supplementary Fig. S1h). This finding suggested that 67NR cells may be able to survive in circulation and establish metastases. To examine this, we introduced 67NR and 4T1 cells into circulation in wild-type mice via tail vein injection. Strikingly, 67NR, which does not metastasise from the mammary fat pad, was able to metastasise to the lung. Lung bioluminescence analysis suggested that 67NR cells formed metastases efficiently similar to 4T1 cells (Fig. 1i–k), and there was no difference in the number of visible nodules between 67NR and 4T1 tumour-bearing mice (Fig. 1l, m). We also observed a similar number of CTCs in mice-bearing 67NR and 4T1 tumours at the end of the experiment (Fig. 1n). Based on these observations, we hypothesised that cells within the TME block 67NR cells from entering the circulation, but not 4T1 cells.

### T cells prevent metastasis

**Fig. 2:**
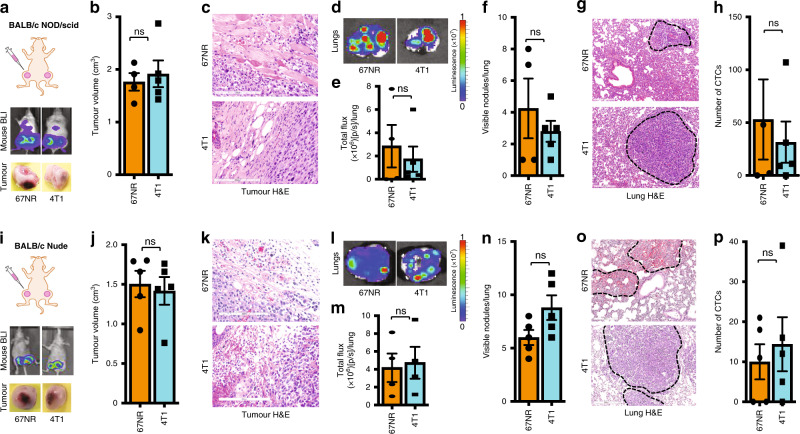
T cells prevent metastasis. In mice with no T, B or NK cells (BALB/c NOD/scid), non-metastatic 67NR breast cancer cells metastasise to the lung from the mammary fat pad similar to metastatic 4T1 cells (**a**–**h**). **a** A cartoon showing the mammary fat pad injection (top). Representative images of whole-body bioluminescence of NOD/scid mice harbouring 67NR and 4T1 tumours (middle) and of tumours excised from these mice (bottom) (*n* = 5). **b** The mean tumour volume of the tumours excised from these mice (*n* = 5). **c** Representative H&E staining of 67NR and 4T1 tumours from BALB/c NOD/scid mice (*n* = 5). Scale bar: 200 µm. **d** Representative bioluminescence images of lungs excised from BALB/c NOD/scid mice harbouring 67NR or 4T1 tumours at the orthotopic mammary fat pad (top). **e** The mean total flux from these lungs (bottom) (*n* = 5). **f** The number of visible lung nodules in 67NR and 4T1 tumour-bearing BALB/c NOD/scid mice (*n* = 5). (**g**) H&E staining of representative lungs from NOD/SCID mice injected with 67NR and 4T1. Scale bar: 200 µm. **h** The number of CTCs isolated from BALB/c NOD/scid mice harbouring 67NR and 4T1 tumours at the mammary fat pad (*n* = 5). In mice with no T cells (BALB/c Nude), non-metastatic 67NR breast cancer cells metastasise to lung from the mammary fat pad similar to metastatic 4T1 cells (**i**–**p**). **i** A cartoon showing the mammary fat pad injection (top). Representative images of whole-body bioluminescence of BALB/c Nude mice harbouring 67NR and 4T1 tumours (middle) and of tumours excised from these mice (bottom) (*n* = 5). **j** The mean tumour volume of the tumours excised from these mice (*n* = 5). **k** Representative H&E staining of 67NR and 4T1 tumours from BALB/c Nude mice (*n* = 5). Scale bar: 200 µm. **l** Representative bioluminescence images of lungs excised from BALB/c Nude mice harbouring 67NR or 4T1 tumours at the orthotopic mammary fat pad (top) and **m** the mean total flux from these lungs (bottom) (*n* = 4). **n** The number of visible lung nodules in 67NR and 4T1 tumour-bearing BALB/c Nude mice (*n* = 5). **o** H&E staining of representative lungs from Nude mice injected with 67NR and 4T1. Scale bar: 300 µm. **p** The number of CTCs isolated from BALB/c Nude mice harbouring 67NR and 4T1 tumours at the mammary fat pad (*n* = 5). Quantitative data are mean ± SEM. Statistical analyses were performed by unpaired two-tailed Student’s *t* test; ns indicates not significant, ^∗^*P* ≤ 0.05, ^∗∗^*P* ≤ 0.01, ^∗∗∗∗^*P* ≤ 0.0001.

To examine whether immune cells from the TME regulate the invasive and metastatic capabilities of 67NR tumour cells, we introduced both 67NR and 4T1 cells into the mammary fat pad of immune-compromised NOD/scid mice. As in the wild-type mice, both 67NR and 4T1 cells grew equally well in the mammary fat pads of these mice as evidenced by similar tumour sizes (Fig. 2a) and tumour volumes (Fig. 2b). However, in contrast to observations in wild-type mice, 67NR cells were highly invasive in the NOD/scid mice (Fig. 2c). The NOD/scid mice injected with 67NR cells developed metastases in the lungs at levels similar to the NOD/scid mice injected with 4T1 cells as evidenced by bioluminescence (Fig. 2d, e) analyses of lung nodules (Fig. 2f, g). In addition, the number of CTCs in 67NR tumour-bearing NOD/scid mice was similar to the number of CTCs in 4T1 tumour-bearing mice (Fig. 2h). Since the NOD/scid mice lack T cells, B cells and have defective natural killer (NK) cells, it was highly likely that one or more of these cell types might be responsible for blocking metastasis of the 67NR cells.

To examine whether T cells regulate metastasis, we introduced 67NR and 4T1 cells into the mammary fat pads of syngeneic BALB/c nude mice that lack T cells; these mice have normal levels of B cells and NK cells. In these mice, 67NR cells formed tumours in the mammary fat pads similar to those formed by 4T1 cells (Fig. 2i, j). Furthermore, in the mice that lack all T-cell subtypes, the 67NR cells were invasive (Fig. 2k) and metastasised to the lungs with an efficiency similar to that of 4T1 cells (Fig. 2l–o). The number of CTCs detectable per 0.5 ml of blood was also similar in the mice lacking T cells injected with 67NR and 4T1 cells (Fig. 2p). Together these findings demonstrate that T cells regulate the metastatic properties of 67NR tumour cells and that 4T1 cells have overcome this inhibition.

### CD8^+^ T cells inhibit metastasis

**Fig. 3:**
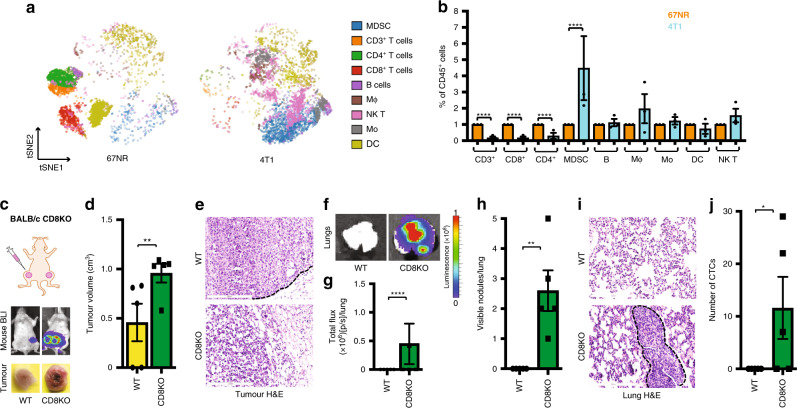
CD8^+^ T cells inhibit metastasis. **a** Representative CyTOF tSNE plots of 67NR and 4T1 tumours grown in wild-type BALB/c mice mammary fat pad. **b** Quantification of CyTOF data of immune populations within CD45^+^ cells from 67NR (orange) and 4T1 (cyan) tumours (*n* = 3). In mice with no CD8^+^ T cells (BALB/c CD8KO), non-metastatic 67NR breast cancer cells metastasise to the lung from the mammary fat pad (**c**–**j**). **c** A cartoon showing the mammary fat pad injection (top). Representative images of whole-body bioluminescence of BALB/c wild-type and BALB/c CD8KO mice harbouring 67NR tumour (middle) and of tumours excised from these mice (bottom) (*n* = 5). **d** The mean tumour volume of the tumours excised from these mice (*n* = 5). **e** Representative H&E staining of tumours from BALB/c wild-type and BALB/c CD8KO mice (*n* = 5). Scale bar: 200 µm. **f** Representative bioluminescence images of lungs excised from BALB/c wild-type and BALB/c CD8KO mice harbouring 67NR at the orthotopic mammary fat pad (top). **g** The mean total flux from these lungs (bottom) (*n* = 5). **h** The number of visible lung nodules in BALB/c wild-type and BALB/c CD8KO tumour-bearing mice (*n* = 5). **i** H&E staining of representative lungs from BALB/c wild-type and BALB/c CD8KO mice. Scale bar: 200 µm. **j** The number of CTCs isolated from BALB/c wild-type and BALB/c CD8KO mice harbouring 67NR tumours at the mammary fat pad (*n* = 5). Quantitative data are mean ± SEM. Statistical analyses were performed by unpaired two-tailed Student’s *t* test for panels **d**, **h** and **j** or by *F* test for panels **b** and **g**; ns indicates not significant, ^∗^*P* ≤ 0.05, ^∗∗^*P* ≤ 0.01, ^∗∗∗∗^*P* ≤ 0.0001.

To identify the T-cell subtype/s that regulate/s metastasis in wild-type mice, we performed CyTOF mass cytometry analyses of 67NR and 4T1 primary tumours grown in wild-type BALB/c mouse mammary fat pads. We found that the CD4^+^ T and CD8^+^ T cells were more abundant and that MDSCs were present in lower numbers in the non-metastatic 67NR tumours than in the metastatic 4T1 tumours (Fig. 3a, b).

To identify the T-cell subtype responsible for spontaneous metastasis, we backcrossed mice lacking CD4^+^ T or CD8^+^ T cells into the BALB/c background and evaluated tumour formation. In the absence of CD4^+^ T cells, the 67NR cells formed primary tumours similar to wild-type mice (Supplementary Fig. S2a–c) also, it did not metastasise (Supplementary Fig. S2d, e). In mice lacking CD8^+^ T cells, however, the 67NR primary tumours were larger than those in wild-type mice (Fig. 3c–e), and, importantly, 67NR cells metastasised to the lungs (Fig. 3f–i) and CTCs were present at higher levels than observed in wild-type mice (Fig. 3j). To further validate the role of CD8^+^ T cells in metastasis, we blocked CD8^+^ T-cell function in wild-type BALB/c mice using a function-blocking antibody (Supplementary Fig. S3a) and injected 67NR cells into the mammary fat pad. As in CD8-knockout mice, in mice treated with the blocking antibody, 67NR cells were highly invasive (Supplementary Fig. S3b) and developed metastatic nodules in the lungs (Supplementary Fig. S3c–e). Together these findings establish that CD8^+^ T cells inhibit metastasis of 67NR tumour cells.

### CXCL4 induces myeloid-derived suppressor cells, which inhibit CD8^+^ T cells and promote metastasis

**Fig. 4:**
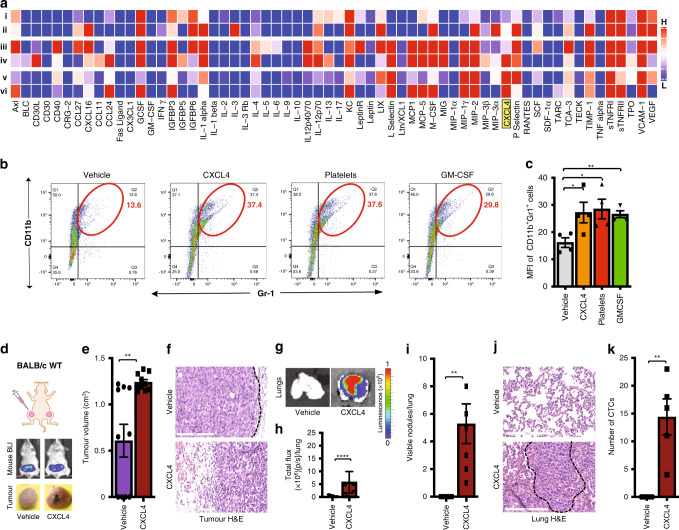
CXCL4 induce myeloid-derived suppressor cells, which inhibit CD8^+^ T cells and promote metastasis. CXCL4 expressed in metastatic tumours makes non-metastatic 67NR tumours to become metastatic in wild-type BALB/c mice (**a**–**c**). **a** Heatmap of mouse cytokine expression array shows CXCL4 (highlighted in yellow) being expressed selectively in metastatic tumours. 67NR cell line (i) and 4T1 cell line (ii); 67NR tumours (iii) and 4T1 tumours (iv) harvested from wild-type BALB/c mice; 67NR tumours (v) and 4T1 tumours (vi) tumours harvested from Nude mice lacking T cells. **b** Flow cytometry analysis for MDSCs in bone marrow cell samples treated with vehicle, CXCL4, platelets or GM-CSF. **c** Quantification of MDSCs in bone marrow cell samples treated with vehicle, CXCL4, platelets or GM-CSF (*n* = 4). In wild-type BALB/c mice, CXCL4 induces non-metastatic 67NR cells to become metastatic from the mammary fat pad to the lung (**d**–**k**). **d** A cartoon showing the mammary fat pad injection (top). Representative images of whole-body bioluminescence of BALB/c wild-type mice harbouring 67NR tumour in the mammary fat pad and treated with vehicle or CXCL4 (middle) and of tumours excised from these mice (bottom) (*n* = 10). **e** The mean tumour volume of the tumours excised from these mice (*n* = 10). **f** Representative H&E staining of tumours excised from these mice (*n* = 5). Scale bar: 200 µm. **g** Representative bioluminescence images of lungs excised from BALB/c wild-type mice harbouring 67NR tumour in the mammary fat pad and treated with vehicle or CXCL4 (top). **h** The mean total flux from these lungs (bottom) (*n* = 10). **i** The number of visible lung nodules in vehicle- or CXCL4-treated tumour-bearing BALB/c wild-type mice (*n* = 7). **j** H&E staining of representative lungs from BALB/c mice harbouring 67NR tumour in the mammary fat pad and treated with vehicle or CXCL4. Scale bar: 200 µm. **k** The number of CTCs isolated from BALB/c wild-type mice harbouring 67NR tumour in the mammary fat pad and treated with vehicle or CXCL4 (*n* = 5). Quantitative data are mean ± SEM. Statistical analyses were performed by unpaired two-tailed Student’s *t* test for panels **c**, **e**, **i** and **k** or by *F* test for panel **h**; ns indicates not significant, ^∗^*P* ≤ 0.05, ^∗∗^*P* ≤ 0.01, ^∗∗∗∗^*P* ≤ 0.0001.

To identify factors responsible for inhibiting CD8^+^ T-cell activity in 4T1 tumours, we analysed the expression of cytokines in protein lysates of non-metastatic 67NR and metastatic 4T1 tumours grown in wild-type mice. Of the cytokines analysed, nine (CD30L, CCL11, CCL24, IGFBP5, IL-4, IL-13, MIP-3ß, CXCL4 and TPO) were significantly more abundant in 4T1 primary tumours than in 67NR primary tumours (Fig. 4aiii, iv and Table 1). We next analysed cytokine expression in protein lysates of 67NR and 4T1 tumours grown in nude mice that lack T cells. Of the nine cytokines differentially expressed in 4T1 and 67NR tumours in wild-type mice, only CXCL4 (also known as platelet-derived factor 4) was expressed in 67NR tumours in the absence of T cells (Fig. 4av and vi). CXCL4 was not expressed by 67NR and 4T1 cells cultured in vitro (Fig. 4ai and ii). Using ELISA, we confirmed that CXCL4 was expressed at lower levels in 67NR tumours than in 4T1 tumours grown in wild-type mice (Supplementary Fig. S4a).

**Table. 1:** Quantification of cytokines in 67NR and 4T1 tumors grown in wild-type or nude mice..

To further interrogate the metastasis-promoting function of CXCL4, we performed a Boyden chamber transwell migration and invasion assay. In this assay, CXCL4 induced both migration and invasion of 67NR cells (Supplementary Fig. S4b, c). To examine whether CXCL4 and platelets directly inhibit CD8^+^ T-cell function, we performed a CD8^+^ T-cell proliferation assay in the presence and absence of CXCL4 or platelets. Neither treatment inhibited CD8^+^ T-cell proliferation (data not shown). Since it is known that MDSCs inhibit the CD8^+^ T-cell function,^8^ we examined whether CXCL4 and platelets induce MDSC production. For this, we incubated monocyte-enriched bone marrow cells with CXCL4 or platelets and quantified MDSCs. Both CXCL4 and platelets increased the generation of MDSCs by approximately twofold (Fig. 4b, c). This induction is similar to that resulting from GM-CSF treatment, which is known to induce MDSC expansion.^44^ To examine whether the MDSCs induced by CXCL4 and platelets inhibits CD8^+^ T-cell activity, we evaluated T-cell proliferation and found that MDSCs were unable to inhibit CD8^+^ T-cell proliferation (Supplementary Fig. S4d). In line with this finding, we found high frequency of MDSCs in mice with metastatic 4T1 tumours (Supplementary Fig. S4e, f), as previously reported.^45^

To investigate the impact of CXCL4 on the development of tumour metastasis, we administered recombinant CXCL4 to wild-type BALB/c mice immediately after the implantation of non-metastatic 67NR tumours in the mammary fat pad and every other day throughout the establishment of tumours. In mice treated with CXCL4, we observed larger primary tumours than in the vehicle-treated group (Fig. 4d–f), and also observed metastasis to the lung in the CXCL4-treated group but not the vehicle-treated group (Fig. 4g–j). We also detected CTCs in the CXCL4-treated group but not in the vehicle-treated group (Fig. 4k). These data clearly demonstrate that CXCL4 promotes metastasis of an otherwise non-invasive tumour type in wildtype mice, even in the presence of CD8^+^ T cells.

### CXCL4 induces metastasis via CXCR3 expressed by tumour cells

CXCL4 functions via the chemokine receptor CXCR3,^27^ which is expressed by various cell types,^46–48^ including tumour cells.^49,50^ Since CXCL4 induced migration (Supplementary Fig. S4b), invasion (Supplementary Fig. S4c) and metastasis of 67NR cells (Fig. 4d–k), we hypothesised that CXCL4 might act through CXCR3 expressed on tumour cells but not on other cell types present within the primary TME. To examine the role of CXCR3 in vivo, we injected 4T1 cells, which express CXCR3, and are metastatic in vivo into the mammary fat pads of wild-type mice. We administered CXCR3 inhibitor AMG487 every day until the termination of the experiment. The CXCR3 inhibitor did not alter primary tumour growth when tumour volumes were compared to vehicle-treated animals (Supplementary Fig. S5a, b). H&E-stained images of these tumours showed that the CXCR3 inhibitor treatment resulted in less invasive tumours (Supplementary Fig. S5c). Treatment with the CXCR3 inhibitor also significantly inhibited metastasis of 4T1 cells to the lungs (Supplementary Fig. S5d, e); visible nodules were reduced by two-fold (Supplementary Fig. S5f). The H&E-stained image shows a reduction in the number of nodules as well (Supplementary Fig. S5g). Importantly, we also observed a threefold decrease in the number of CTCs upon CXCR3 inhibitor treatment compared to the vehicle-treated mice (Supplementary Fig. S5h).

### CD8^+^ T cells inversely correlate with platelets and predict metastasis-free survival

**Fig. 5:**
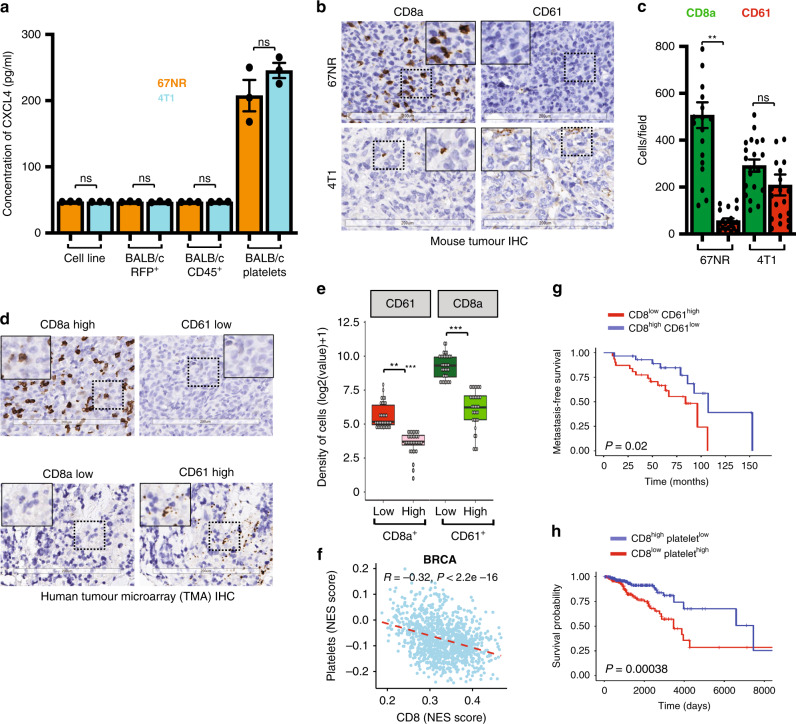
CD8^+^ T cells inversely correlate with platelets and predict metastasis-free survival. **a** CXCL4 is selectively expressed in platelets (CD61^+^) and not by cultured 67NR and 4T1 cells, or the 67NR or 4T1 tumour cells isolated from BALB/c mouse harbouring these tumours, or by CD45^+^ fractions from these mice (*n* = 3). **b** Representative images of 67NR and 4T1 tumours grown in wild-type BALB/c mice stained with CD8a and CD61 (platelets). Scale bars: 200 µm. **c** Quantification of immunohistochemistry of 67NR and 4T1 tumours grown in wild-type BALB/c mice stained with CD8a and CD61(*n* = 20, analysed five areas per tumour, a total of four tumours). **d** Representative images of human triple-negative breast cancer tissues stained with CD8a and CD61 (platelets), scale bars, 200 µm. **e** Quantification of immunohistochemistry of human triple-negative breast cancer tissues stained with CD8a and CD61 (*n* = 68). **f** Negative correlation between platelets and CD8^+^ T cells in breast cancer using TCGA data. **g** Metastasis-free survival probability of patients with a low frequency of CD8^+^ T cells and high frequency of platelets (CD61^+^ cells) relative to breast cancer patients with a high frequency of CD8^+^ T cells and low frequency of CD61^+^ cells (*n* = 68) in breast tissue TMA. **h** Overall survival probability of breast cancer patients with high levels of CD8^+^ T cells and low levels of platelets compared to patients with low levels of CD8^+^ T cells and high levels of platelets (data obtained from TCGA, *n* = 1093). Quantitative data are mean ± SEM. Statistical analyses were performed by unpaired two-tailed Student’s *t* test for panels **a** and **c** or by log-rank test for panel e; ns indicates not significant, ^∗^*P* ≤ 0.05, ^∗∗^*P* ≤ 0.01, ^∗∗∗^*P* ≤ 0.001, ^∗∗∗∗^*P* ≤ 0.0001.

To identify the cell type that produces CXCL4, we isolated platelets, which are known to produce CXCL4,^30,31^ CD45^+^ hematopoietic cells and tumour cells from 67NR and 4T1 tumours grown in wild-type BALB/c mice. Analysis of CXCL4 expression by ELISA in these populations from each tumour showed that CXCL4 was expressed in platelets but not in tumour cells or CD45^+^ hematopoietic cells (Fig. 5a). We hypothesised that non-metastatic 67NR tumours have a lower number of CXCL4-producing platelets than do metastatic tumours and that the presence of platelets should inversely correlate with the number of CD8^+^ T cells. To evaluate these hypotheses, we performed immunocytochemistry of 67NR and 4T1 tumours isolated from wild-type BALB/c mice using antibodies to CD8a and CD61 to stain CD8^+^ T cells and platelets, respectively. We observed a high frequency of CD8^+^ T cells and a low frequency of platelets in non-metastatic 67NR tumours and the inverse in 4T1 tumours (Fig. 5b and quantified in c). In line with our observations in mice, we found a strong positive correlation between platelets and MDSCs in human breast cancer tumour samples (Spearman’s correlation, *r* = 0.73, *P* < 10^−23^) and other cancer types (Supplementary Fig. S6a). We also observed a strong negative correlation between levels of CD8^+^ T cells and MDSCs (Supplementary Fig. S6a) and between CD8^+^ T cells and platelets in breast tumours (*r* = −0.14, *P* = 1.5 × 10^−6^ and *r* = −0.3, *P* = 3.33 × 10^−24^, respectively) and across cancer types.

To determine whether these observations are relevant in human disease, we analysed a tissue microarray from the Breast Tumor Bank with 180 human triple-negative breast tumour tissues for the presence of CD8^+^ T cells and platelets by immunocytochemistry. In agreement with our mouse tumour tissue data, patients with high platelet counts had low numbers of CD8^+^ T cells, and patients with low platelet counts had high numbers of CD8^+^ T cells (Fig. 5d and quantified in e).

We also analysed TCGA transcriptomics data for correlations between CXCL4-producing platelets, MDSCs, and CD8^+^ T cells using the Gene Set Variation Analysis method^39^ with immune marker gene sets previously described.^40,41^ We observed a striking negative correlation between CD8^+^ T cells and CXCL4-producing platelets in breast tumours (*R* = −0.32, *P* < 2.2 × 10^−16^, Fig. 5f, Table 2) as well as a strong positive correlation between platelets and MDSCs (*R* = 0.67, *P* < 2.2 × 10^−16^) across cancer types (Table 2).

**Table. 2:** Correlations between CD8+ T cells and platelet abundances and between platelet and MDSC frequencies in various cancers represented in TCGA.

The red color represents a negative correlation, and the green color represents a positive correlation.

By analysis of a tissue microarray from the Breast Tumor Bank with samples from 180 patients with breast cancer for whom clinical data are available, we found that patients with high levels of platelets and low levels of CD8^+^ T cells had decreased metastasis-free survival than those with the low levels of platelets and high CD8^+^ T cells (HR = 2.84 (1.14–7.08) log-rank *P* = 0.02, Fig. 5g). We also analysed the TCGA data and found that the breast cancer patients with low frequencies of CD8^+^ T cells and high frequencies of platelets had much lower overall survival rates than patients with high frequencies of CD8^+^ T cells and low frequencies of platelets (HR = 2.511 (1.48–4.25), log-rank *P* = 0.00038, Fig. 5h, Table 3). Similarly, the overall survival of breast cancer patients with low frequencies of CD8^+^ T cells and high frequencies of MDSCs had lower survival compared to patients with high frequencies of CD8^+^ T cells and low frequencies of MDSCs (log-rank test, HR = 3.37 (1.88–6.05), *P* < 0.0001 (Supplementary Fig. S6b). Further, breast cancer patients with high levels of platelets and MDSCs had a very low survival rate compared to those with low levels of platelets and MDSCs (log-rank test, HR = 1.78 (1.14–2.79), *P* = 0.01) (Supplementary Fig. S6c). Finally, we analysed the overall survival of breast cancer patients using TCGA data and found patients with low CD8^+^ T-cell level and high levels of platelets and MDSCs had low survival probability compared to those with high levels of CD8^+^ T cells and low levels of platelets and MDSCs (log-rank test, HR = 5.09 (2.04–12.65), *P* = 0.00011) (Supplementary Fig. S6d). Similar results were obtained when frequencies of platelets, MDSCs, and CD8^+^ T cells were correlated with the survival of patients with other types of cancer (Supplementary Fig. S6e).

**Table. 3:** Hazard ratio for overall survival of cancer patients with low CD8+ T cells and high platelets.

The green color represents positive correlation.

Together, our findings provide strong evidence that the presence of high frequencies of platelets and low frequencies of CD8^+^ T cells within the primary tumour is predictive of poor prognosis for patients with many types of cancer.

## Discussion

The intricate interaction between tumour cells and immune cells determines whether or not tumours metastasise.^7–16,51^ In our study, we demonstrate for the first time that the platelets secrete CXCL4 to induce MDSC production and that MDSCs, in turn, inhibits the function of CD8^+^ T cells. This facilitates the escape of tumour cells from the primary tumour and into circulation resulting in the establishment of metastasis. Significant platelet involvement in the development and progression of cancers was first clinically documented almost 150 years ago.^52^ Increased platelet counts (thrombocythemia) are a hallmark of advanced cancers and decreased survival of cancers of the breast and lung.^53^ Mechanistically, platelets induce the epithelial–mesenchymal transition, promote intravasation of tumour cells and enhance the survival of circulating tumour cells by encapsulation, eventually promoting extravasation at a distant site and metastasis.^28,29,54–59^ Platelets are also known to promote immune tolerance by secreting TGF-ß1, which stimulates the innate immune response and inhibits the adaptive immune system.^58^

We observed that metastatic tumours have higher frequencies of platelets and lower numbers of CD8^+^ T cells than non-metastatic tumours, suggesting that the interplay between CD8^+^ T cells and platelets influences metastasis. We established that platelets can alter the function and fate of T cells within the tumour microenvironment and that the platelet-MDSC-CD8^+^ T-cell axis plays an important role in determining the ability of breast tumours to metastasise. CD8^+^ T cells were previously only indirectly linked with metastasis;^60,61^ this study is the first in vivo demonstration that CD8^+^ T cells directly inhibit metastasis. During CD8^+^ T-cell exhaustion, the cells become dysfunctional, produce a lower amount of cytokines, and express markers of apoptosis.^62–64^ Exhausted T cells also express immune checkpoint molecules, including PD-1, TIM-3 and CTLA‐4.^21^ MDSCs are known to induce expression of exhaustion markers on CD8^+^ T cells in patients with myelodysplastic syndromes^65^ and during the immunotolerance induced by the hepatitis B virus.^66^ We believe that the CD8^+^ T cells may actively clear platelets in non-metastatic tumours similar to that observed in the liver.^67^ Since CXCL4 is primarily produced by platelets,^30,31^ we hypothesised that the expression of CXCL4 depends on the presence of platelets within the tumour. The inverse relationship we observed between frequencies of platelets and CD8^+^ T cells in various immune backgrounds suggests that platelet function may be regulated by the presence of CD8^+^ T cells. We speculate that CD8^+^ T cells actively inhibit and/or eliminate platelets from the TME, and, as a result, the tumours with high infiltration of CD8^+^ T cells have fewer platelets and hence, less CXCL4. In contrast, tumours with low numbers of CD8^+^ T cells will have higher levels of platelets, which secrete CXCL4, promoting invasion and metastasis by generating MDSCs, and inhibiting CD8^+^ T-cell function. CXCL4 functions via the chemokine receptor CXCR3,^27^ which is expressed by various cell types, including tumour cells. Our data suggest that CXCL4 acts on tumour cells via CXCR3 to regulate their migration and invasion.

We demonstrated that CXCL4 secreted by platelets induces the production of MDSCs, which in turn negatively regulate CD8^+^ T-cell function, as shown by the induction of exhaustion markers in a mouse model. We demonstrated a link between platelet-derived CXCL4 and CD8^+^ T cells through MDSCs, but it is likely that other CXCL4-dependent mechanisms, such as effects on endothelial cells and fibroblast activation, also alter CD8^+^ T-cell infiltration into tumours. Analyses of TCGA data on breast and various types of cancer revealed that patients with high levels of platelets were more likely to develop metastases with poor overall survival than patients with high levels of CD8^+^ T cells and low platelets. These findings suggest that inhibiting CXCL4 may promote the recruitment of CD8^+^ T cells and inhibit metastasis.

## Supplementary information

Supplementary information

Figure S1

Figure S2

Figure S3

Figure S4

Figure S5

Figure S6

## Data Availability

Data are available from S.A.M. upon a reasonable request.

## References

[1] Sengupta R, Honey K (2018). AACR Cancer Progress Report 2018: harnessing research discoveries for patient benefit. Clin. Cancer Res..

[2] Gupta GP, Massague J (2006). Cancer metastasis: building a framework. Cell.

[3] Hanahan D, Weinberg RA (2011). Hallmarks of cancer: the next generation. Cell.

[4] Pantel K, Brakenhoff RH (2004). Dissecting the metastatic cascade. Nat. Rev. Cancer.

[5] Psaila B, Lyden D (2009). The metastatic niche: adapting the foreign soil. Nat. Rev. Cancer.

[6] Zetter BR (1998). Angiogenesis and tumor metastasis. Annu Rev. Med..

[7] Coussens LM, Werb Z (2002). Inflammation and cancer. Nature.

[8] Gabrilovich DI, Nagaraj S (2009). Myeloid-derived suppressor cells as regulators of the immune system. Nat. Rev. Immunol..

[9] Gonzalez H, Hagerling C, Werb Z (2018). Roles of the immune system in cancer: from tumor initiation to metastatic progression. Genes Dev..

[10] Kitamura T, Qian BZ, Pollard JW (2015). Immune cell promotion of metastasis. Nat. Rev. Immunol..

[11] Labelle M, Hynes RO (2012). The initial hours of metastasis: the importance of cooperative host-tumor cell interactions during hematogenous dissemination. Cancer Discov..

[12] Marusyk A, Almendro V, Polyak K (2012). Intra-tumour heterogeneity: a looking glass for cancer?. Nat. Rev. Cancer.

[13] Ostrand-Rosenberg S, Beury DW, Parker KH, Horn LA (2020). Survival of the fittest: how myeloid-derived suppressor cells survive in the inhospitable tumor microenvironment. Cancer Immunol. Immunother..

[14] Pages F, Galon J, Dieu-Nosjean MC, Tartour E, Sautes-Fridman C, Fridman WH (2010). Immune infiltration in human tumors: a prognostic factor that should not be ignored. Oncogene.

[15] Quail DF, Joyce JA (2013). Microenvironmental regulation of tumor progression and metastasis. Nat. Med..

[16] Steeg PS (2016). Targeting metastasis. Nat. Rev. Cancer.

[17] Binnewies M, Roberts EW, Kersten K, Chan V, Fearon DF, Merad M (2018). Understanding the tumor immune microenvironment (TIME) for effective therapy. Nat. Med..

[18] Pardoll DM (2012). The blockade of immune checkpoints in cancer immunotherapy. Nat. Rev. Cancer.

[19] Rosenberg SA (2014). Decade in review-cancer immunotherapy: entering the mainstream of cancer treatment. Nat. Rev. Clin. Oncol..

[20] Hodi FS, O’Day SJ, McDermott DF, Weber RW, Sosman JA, Haanen JB (2010). Improved survival with ipilimumab in patients with metastatic melanoma. N. Engl. J. Med..

[21] Sharma P, Allison JP (2015). Immune checkpoint targeting in cancer therapy: toward combination strategies with curative potential. Cell.

[22] Baitsch L, Baumgaertner P, Devevre E, Raghav SK, Legat A, Barba L (2011). Exhaustion of tumor-specific CD8(+) T cells in metastases from melanoma patients. J. Clin. Investig..

[23] DeNardo DG, Barreto JB, Andreu P, Vasquez L, Tawfik D, Kolhatkar N (2009). CD4(+) T cells regulate pulmonary metastasis of mammary carcinomas by enhancing protumor properties of macrophages. Cancer Cell.

[24] Tian L, Goldstein A, Wang H, Ching Lo H, Sun Kim I, Welte T (2017). Mutual regulation of tumour vessel normalization and immunostimulatory reprogramming. Nature.

[25] Badovinac VP, Harty JT (2000). Adaptive immunity and enhanced CD8+ T cell response to Listeria monocytogenes in the absence of perforin and IFN-gamma. J. Immunol..

[26] Menter DG, Tucker SC, Kopetz S, Sood AK, Crissman JD, Honn KV (2014). Platelets and cancer: a casual or causal relationship: revisited. Cancer Metastasis Rev..

[27] Vandercappellen J, Van Damme J, Struyf S (2011). The role of the CXC chemokines platelet factor-4 (CXCL4/PF-4) and its variant (CXCL4L1/PF-4var) in inflammation, angiogenesis and cancer. Cytokine Growth Factor Rev..

[28] Haemmerle M, Taylor ML, Gutschner T, Pradeep S, Cho MS, Sheng J (2017). Platelets reduce anoikis and promote metastasis by activating YAP1 signaling. Nat. Commun..

[29] Labelle M, Begum S, Hynes RO (2011). Direct signaling between platelets and cancer cells induces an epithelial-mesenchymal-like transition and promotes metastasis. Cancer Cell.

[30] Bruns I, Lucas D, Pinho S, Ahmed J, Lambert MP, Kunisaki Y (2014). Megakaryocytes regulate hematopoietic stem cell quiescence through CXCL4 secretion. Nat. Med..

[31] Kowalska MA, Rauova L, Poncz M (2010). Role of the platelet chemokine platelet factor 4 (PF4) in hemostasis and thrombosis. Thromb. Res..

[32] Faustino-Rocha A, Oliveira PA, Pinho-Oliveira J, Teixeira-Guedes C, Soares-Maia R, da Costa RG (2013). Estimation of rat mammary tumor volume using caliper and ultrasonography measurements. Lab. Anim..

[33] Acharya S, Yao J, Li P, Zhang C, Lowery FJ, Zhang Q (2019). Sphingosine kinase 1 signaling promotes metastasis of triple-negative breast cancer. Cancer Res..

[34] Parra ER, Behrens C, Rodriguez-Canales J, Lin H, Mino B, Blando J (2016). Image analysis-based assessment of PD-L1 and tumor-associated immune cells density supports distinct intratumoral microenvironment groups in non-small cell lung carcinoma patients. Clin. Cancer Res..

[35] Schneider CA, Rasband WS, Eliceiri KW (2012). NIH Image to ImageJ: 25 years of image analysis. Nat. Methods.

[36] McCarthy RL, Mak DH, Burks JK, Barton MC (2017). Rapid monoisotopic cisplatin based barcoding for multiplexed mass cytometry. Sci. Rep..

[37] McCarthy RL, Duncan AD, Barton MC (2017). Sample preparation for mass cytometry analysis. J. Vis. Exp..

[38] Schroder M, Krotschel M, Conrad L, Naumann SK, Bachran C, Rolfe A (2018). Genetic screen in myeloid cells identifies TNF-alpha autocrine secretion as a factor increasing MDSC suppressive activity via Nos2 up-regulation. Sci. Rep..

[39] Hanzelmann S, Castelo R, Guinney J (2013). GSVA: gene set variation analysis for microarray and RNA-seq data. BMC Bioinforma..

[40] Angelova M, Charoentong P, Hackl H, Fischer ML, Snajder R, Krogsdam AM (2015). Characterization of the immunophenotypes and antigenomes of colorectal cancers reveals distinct tumor escape mechanisms and novel targets for immunotherapy. Genome Biol..

[41] van Velzen JF, Laros-van Gorkom, B. A, Pop GA, van Heerde WL (2012). Multicolor flow cytometry for evaluation of platelet surface antigens and activation markers. Thromb. Res..

[42] Aslakson CJ, Miller FR (1992). Selective events in the metastatic process defined by analysis of the sequential dissemination of subpopulations of a mouse mammary tumor. Cancer Res..

[43] Lelekakis M, Moseley JM, Martin TJ, Hards D, Williams E, Ho P (1999). A novel orthotopic model of breast cancer metastasis to bone. Clin. Exp. Metastasis.

[44] Marigo I, Bosio E, Solito S, Mesa C, Fernandez A, Dolcetti L (2010). Tumor-induced tolerance and immune suppression depend on the C/EBPbeta transcription factor. Immunity.

[45] Welte T, Kim IS, Tian L, Gao X, Wang H, Li J (2016). Oncogenic mTOR signalling recruits myeloid-derived suppressor cells to promote tumour initiation. Nat. Cell Biol..

[46] Garcia-Lopez MA, Sanchez-Madrid F, Rodriguez-Frade JM, Mellado M, Acevedo A, Garcia MI (2001). CXCR3 chemokine receptor distribution in normal and inflamed tissues: expression on activated lymphocytes, endothelial cells, and dendritic cells. Lab. Investig..

[47] Groom JR, Luster AD (2011). CXCR3 in T cell function. Exp. Cell Res..

[48] Perney P, Turriere C, Portales P, Rigole H, Psomas C, Blanc F (2009). CXCR3 expression on peripheral CD4+ T cells as a predictive marker of response to treatment in chronic hepatitis C. Clin. Immunol..

[49] Jin J, Zhang Z, Wang H, Zhan Y, Li G, Yang H (2018). CXCR3 expression in colorectal cancer cells enhanced invasion through preventing CXCR4 internalization. Exp. Cell Res..

[50] Robledo MM, Bartolome RA, Longo N, Rodriguez-Frade JM, Mellado M, Longo I (2001). Expression of functional chemokine receptors CXCR3 and CXCR4 on human melanoma cells. J. Biol. Chem..

[51] Smith HA, Kang Y (2013). The metastasis-promoting roles of tumor-associated immune cells. J. Mol. Med..

[52] Trousseau, A. Phlegmatia alba dolens. in *Clinique Medicale de l’Hotel-Dieu de Paris*, (ed. J.-B. Baillière et fils) Vol. 3, 654–712 (Lectures on Clinical Medicine at the Hotel-Dieu, 1865).

[53] Sylman, J. L., Mitrugno, A., Tormoen, G. W., Wagner, T. H., Mallick, P. & McCarty, O. J. T. Platelet count as a predictor of metastasis and venous thromboembolism in patients with cancer. *Converg. Sci. Phys. Oncol.* **3**, 023001 (2017). 10.1088/2057-1739/aa6c05 PMC5658139 29081989

[54] Gasic GJ, Gasic TB, Galanti N, Johnson T, Murphy S (1973). Platelet-tumor-cell interactions in mice. The role of platelets in the spread of malignant disease. Int. J. Cancer.

[55] Gay LJ, Felding-Habermann B (2011). Contribution of platelets to tumour metastasis. Nat. Rev. Cancer.

[56] Honn KV, Tang DG, Crissman JD (1992). Platelets and cancer metastasis: a causal relationship?. Cancer Metastasis Rev..

[57] Janowska-Wieczorek A, Wysoczynski M, Kijowski J, Marquez-Curtis L, Machalinski B, Ratajczak J (2005). Microvesicles derived from activated platelets induce metastasis and angiogenesis in lung cancer. Int. J. Cancer.

[58] Li, Z., Riesenberg, B., Metelli, A., Li, A. & Wu, B.X. in *Platelets*, fourth edn. (eds. Michelson, A., Cattaneo, M., Frelinger, A., & Newman, P.) Vol. 30, 547–561 (Academic Press, 2019).

[59] Palumbo JS, Talmage KE, Massari JV, La Jeunesse CM, Flick MJ, Kombrinck KW (2005). Platelets and fibrin(ogen) increase metastatic potential by impeding natural killer cell-mediated elimination of tumor cells. Blood.

[60] Chen L, Gibbons DL, Goswami S, Cortez MA, Ahn YH, Byers LA (2014). Metastasis is regulated via microRNA-200/ZEB1 axis control of tumour cell PD-L1 expression and intratumoral immunosuppression. Nat. Commun..

[61] Visus C, Wang Y, Lozano-Leon A, Ferris RL, Silver S, Szczepanski MJ (2011). Targeting ALDH(bright) human carcinoma-initiating cells with ALDH1A1-specific CD8(+) T cells. Clin. Cancer Res..

[62] Fourcade J, Sun Z, Benallaoua M, Guillaume P, Luescher IF, Sander C (2010). Upregulation of Tim-3 and PD-1 expression is associated with tumor antigen-specific CD8+ T cell dysfunction in melanoma patients. J. Exp. Med..

[63] Kim HD, Song GW, Park S, Jung MK, Kim MH, Kang HJ (2018). Association between expression level of PD1 by tumor-infiltrating CD8(+) T cells and features of hepatocellular carcinoma. Gastroenterology.

[64] Taghiloo S, Allahmoradi E, Tehrani M, Hossein-Nataj H, Shekarriz R, Janbabaei G (2017). Frequency and functional characterization of exhausted CD8(+) T cells in chronic lymphocytic leukemia. Eur. J. Haematol..

[65] Tao J, Han D, Gao S, Zhang W, Yu H, Liu P (2020). CD8(+) T cells exhaustion induced by myeloid-derived suppressor cells in myelodysplastic syndromes patients might be through TIM3/Gal-9 pathway. J. Cell Mol. Med..

[66] Kong X, Sun R, Chen Y, Wei H, Tian Z (2014). gammadeltaT cells drive myeloid-derived suppressor cell-mediated CD8+ T cell exhaustion in hepatitis B virus-induced immunotolerance. J. Immunol..

[67] Qiu J, Liu X, Li X, Zhang X, Han P, Zhou H (2016). CD8(+) T cells induce platelet clearance in the liver via platelet desialylation in immune thrombocytopenia. Sci. Rep..

