# Supplementary material for: CD8+ T cells inhibit metastasis and CXCL4 regulates its function
Source: Br J Cancer. 2021 Apr 1;125(2):176–89. doi: 10.1038/s41416-021-01338-5 (PMC8292398; doi:10.1038/s41416-021-01338-5)
Supplement: Supplementary file 1 — Supplementary information [file 41416_2021_1338_MOESM1_ESM.docx]

**SUPPLEMENTARY INFORMATION**

**SUPPLEMENTARY FIGURES**

**Supplementary Figure S1, Related to Figure 1. Non-metastatic 67NR develop extensive metastasis similar to metastatic 4T1 cells when introduced via tail vein in wildtype BALB/c mice.** **(a)** Growth curve of 67NR and 4T1 cells over a period of 96 hours *in vitro* culture. **(b)** Quantification of soft agar colony formation by 67NR and 4T1 cells (n=3). **(c)** Quantification of acini formed by 67NR and 4T1 cells (n=3). **(d)** Representative images of CD31 staining of 67NR and 4T1 tumors grown in mammary glands of wildtype BALB/c mice. Scale bar, 200 µm. **(e)** Quantification of CD31 staining of 67NR and 4T1 tumors grown in mammary glands of wildtype BALB/c mice (n=5 per group). **(f)** Representative images of Ki67 staining of 67NR and 4T1 tumors grown in mammary glands of wildtype BALB/c mice. Scale bar, 200 µm. **(g)** Quantification of Ki67 staining of 67NR and 4T1 tumors grown in mammary glands of wildtype BALB/c mice (n=5 per group). **(h)** Quantification of survival of 67NR and 4T1 cells in suspension culture (n=3). Quantitative data are mean ± SEM. Statistical analyses were performed by unpaired two-tailed Student's t-test; ns indicates not significant.

**Supplementary Figure S2, Related to Figure 3.** **CD4^+^ T cells are not involved in regulating the metastasis of 67NR cells.** In mice with no CD4^+^ T cells (BALB/c CD4KO), non-metastatic 67NR breast cancer cells remain non-metastatic from the mammary fat pad. **(a)** A cartoon showing the mammary fat pad injection (top). Representative images of whole-body bioluminescence of BALB/c wildtype and BALB/c CD4KO mice harboring 67NR tumor and of tumors excised from these mice (n=5). **(b)** The mean tumor volume of the tumors excised from these mice (n=5). **(c)** H&E staining of representative tumors from BALB/c wildtype and BALB/c CD4KO mice injected with 67NR. Scale bar, 200 µm. **(d)** Representative bioluminescence images of lungs excised from BALB/c wildtype and BALB/c CD4KO mice harboring 67NR at the orthotopic mammary fat-pad (n=5). **(e)** H&E staining of representative lungs from BALB/c wildtype and BALB/c CD4KO mice injected with 67NR. Scale bar, 200 µm. Quantitative data are mean ± SEM. Statistical analyses were performed by unpaired two-tailed Student's t-test; ns indicates not significant.

**Supplementary Figure S3, Related to Figure 3. CD8^+^ T cells regulate metastasis of tumor cells. (a)** FACS analysis of mice injected with anti-CD8 antibody. **(b)** Representative H&E staining of 67NR primary mammary tumor grown in wildtype mice treated with anti-CD8 antibody or anti-IgG control. Scale bar, 200 µm. **(c)** Representative images of bioluminescence of lungs removed from wildtype mice harboring 67NR primary mammary tumors treated with anti-CD8 antibody or anti-IgG control. **(d)** Quantification of total flux from the lungs of wildtype mice bearing 67NR primary mammary tumors treated with anti-CD8 antibody or anti-IgG control (n=4 per group). **(e)** Representative H&E staining of 67NR lungs from mice injected with anti-CD8 antibody or anti-IgG control. Scale bar, 200 µm. Quantitative data are mean ± SEM. Statistical analyses were performed by unpaired two-tailed Student's t-test; ns indicates not significant.

**Supplementary Figure S4, Related to Figure 4.** **CXCL4 and platelets inhibit CD8^+^ T cell function by inducing MDSC expansion.** **(a)** Concentration of CXCL4 in 67NR and 4T1 cells in culture and in tumors harvested from wildtype mice, Nude mice and NOD/scid mice determined using ELISA (n=3 per group). **(b)** Quantification of 67NR and 4T1 cell migration in the presence of CXCL4 (measure from 10 fields) (n=2). **(c)** Quantification of 67NR and 4T1 cell invasion in the presence of CXCL4 (measure from 10 fields) (n=2). **(d)** Analysis of CD8^+^ T cell proliferation in bone marrow cell samples treated with vehicle, CXCL4, platelets, or GM-CSF for 4 days. **(e)** Representative FACS quantification of the percentage of MDSCs in 67NR and 4T1 tumors grown in wildtype mice. **(f)** Quantification of median fluorescent intensities (MFI) of MDSC population in 67NR and 4T1 tumors grown in wildtype mice (n=6 per group). Quantified data are mean ± SEM. Statistical analyses were performed by unpaired two-tailed Student's t-test for panels a, b, c and f; ns indicates not significant, ∗∗p ≤ 0.01, ∗∗∗∗p ≤ 0.0001.

**Supplementary Figure S5, Related to Figure 4. CXCL4 induces metastasis via CXCR3 on tumor cells. (a)** Representative bioluminescence images of whole body of wild-type mice harboring 4T1 primary mammary tumors and treated with AMG487 or vehicle (top), and images of the tumors excised from these mice (bottom panel). **(b)** Quantification of 4T1 primary tumor volumes from wild-type mice treated with vehicle or AMG487 (n=5). **(c)** Representative H&E staining of 4T1 tumors from mice treated with vehicle or AMG487. Scale bar, 200 µm. **(d)** Representative bioluminescence images of 4T1 tumor-bearing mice treated with vehicle or AMG487. **(e)** Total flux in photons per second from the lungs of 4T1 tumor-bearing mice treated with vehicle or AMG487 (n=5). **(f)** Quantification of visible nodules in lungs of 4T1 tumor-bearing mice treated with vehicle or AMG487 (n=5). **(g)** Representative H&E-stained images of lungs from 4T1 tumor-bearing mice treated with vehicle or AMG487. Scale bar, 200 µm. **(h)** Quantification of CTCs isolated from 4T1 tumor-bearing mice treated with vehicle or AMG487 (n=5). Quantitative data are mean ± SEM. Statistical analyses were performed by unpaired two-tailed Student’s t-test; ns indicates not significant, ∗p ≤ 0.05, ∗∗p ≤ 0.01, ∗∗∗p ≤ 0.001, ∗∗∗∗p ≤ 0.0001.

**Supplementary Figure S6, Related to Figure 5.** **Correlations between CD8^+^ T cells, platelets and MDSCs.** **(a)** Spearman’s correlation between MDSC and platelets, CD8^+^ T cell and MDSC and CD8^+^ T cell and platelet frequencies across various cancers (data obtained from TCGA). **(b)** Survival probability of breast cancer patients with high levels of CD8^+^ T cells and low levels of MDSCs compared to patients with low levels of CD8^+^ T cells and high levels of MDSCs (data obtained from TCGA). **(c)** Survival probability of breast cancer patients with low levels of platelets and MDSCs compared to patients with high levels of these cells (data obtained from TCGA). **(d)** Survival probability of breast cancer patients with high levels of CD8^+^ T cells, low levels of platelets, and low levels of MDSCs compared to patients with low levels of CD8^+^ T cells, high levels of platelets, and high levels of MDSCs (data obtained from TCGA). **(e)** Survival analysis of patients based on Cox proportional hazard regression for various cancers with low levels of CD8^+^ T cells and high levels of platelets (red), low levels of CD8^+^ T cells and high levels of MDSCs (yellow), and high levels of platelets and high levels of MDSCs (blue) (data obtained from TCGA).

**SUPPLEMENTARY TABLES**

**Table 1, Related to Figure 4.** **Quantification of cytokines in protein lysates of non-metastatic 67NR and metastatic 4T1 tumors grown in wildtype mice or nude mice that lack T cells.** The intensity of the signal from the membrane was quantified using ImageJ.

**Table 2, Related to Figure 5. Correlations between CD8^+^ T cells and platelet abundances and between platelet and MDSC frequencies in various cancers represented in TCGA.** Cancer cohorts used for the analysis were adrenocortical carcinoma (ACC), bladder urothelial carcinoma (BLCA), breast invasive carcinoma (BRCA), cervical and endocervical cancers (CESC), cholangiocarcinoma (CHOL), colon and rectal cancer (CRC), lymphoid neoplasm diffuse large B cell lymphoma (DLBC), esophageal carcinoma (ESCA), glioblastoma multiforme (GBM), head and neck squamous cell carcinoma (HNSC), kidney chromophobe (KICH), kidney renal clear cell carcinoma (KIRC), kidney renal papillary cell carcinoma (KIRP), acute myeloid leukaemia (LAML), brain lower grade glioma (LGG), liver hepatocellular carcinoma (LIHC), lung adenocarcinoma (LUAD), lung squamous cell carcinoma (LUSC), mesothelioma (MESO), ovarian serous cystadenocarcinoma (OV), pancreatic adenocarcinoma (PAAD), pheochromocytoma and paraganglioma (PCPG), prostate adenocarcinoma (PRAD), sarcoma (SARC), skin cutaneous melanoma (SKCM), stomach adenocarcinoma (STAD), testicular germ cell tumours (TGCT), thyroid carcinoma (THCA), thymoma (THYM), uterine corpus endometrial carcinoma (UCEC), uterine carcinosarcoma (UCS), uveal melanoma (UVM). The number of patients in each cohort are listed in Table 3.

**Table 3, Related to Figure 5. Hazard ratio for overall survival of cancer patients with low CD8^+^ T cells and high platelets.** Abbreviations are listed in Table 2.

**SUPPLEMENTARY METHODS**

***In vivo* bioluminescence analysis**

D-Luciferin (Fisher Scientific, catalog number PI88294) was dissolved in PBS at 15 mg/ml and sterilized through a 0.2-µm filter. Mice were given 150 mg D-luciferin per kg body weight subcutaneously. Within 15 minutes of dosing, animals were subjected to imaging in the IVIS Spectrum. The bioluminescence images were analyzed using the Living Image software 64-bit (PerkinElmer).

**Isolation of circulating tumor cells from the blood of experimental mice**

Immediately after the sacrifice of the mice, about 200 μl of blood was harvested via cardiac puncture into EDTA-coated blood collection tubes (Fisher Scientific, catalog number 02-683-99D) that were stored on ice. The blood was then spun down at 200 x g for 5 minutes, and each pellet was resuspended in 1 ml ACK-lysing buffer (Life Technologies, catalog number A10492-01) and further incubated for 5 minutes at room temperature to rupture the red blood cells. The lysis reaction was neutralized by adding 5 ml DMEM-F12 medium containing 10% FBS, and the cells were spun down at 200 x g for 5 minutes. Cells were washed once with PBS, resuspended in DMEM-F12 medium with 10% FBS and 5% penicillin/streptomycin, and cultured on either 6-cm or 10-cm tissue culture dishes based on the number of CTCs anticipated. The cell culture medium was changed daily to remove dead cells. CTCs, if present, were visible as colonies within 7 days. The RFP-positive colonies (originating from the labeled 67NR or 4T1 cells injected into mice) were quantified by fluorescence microscopy using a Zeiss microscope.

**Cell viability and survival in suspension assay**

Cells (1x10^4^) were grown in 2D cultures for 0, 12, 24, 48, 72, and 96 hours, and viable cells were counted using Trypan blue stain using a hemocytometer. To determine viability in suspension, cells were grown in 1% agarose, and live cells present at 0, 1, 3, 6, 9 and 12 hours were counted on a hemocytometer after Trypan blue staining.

**Soft agar colony formation assay**

To evaluate the tumorigenic capacity of the 67NR and 4T1 cells, cells were subjected to an anchorage-independent growth assay, and their ability to form colonies in soft agar was measured. We suspended 2.5x10^3^ cells in 0.7% top agar and placed aliquots onto the base agar of 0.8% sea plaque agarose (Lonza, catalog number 50101). After the solidification of the top agar, 500 µl of fresh complete DMEM/F12 medium was added on top of the top agar, and the plates were incubated for 14 days at 37 °C. The cells were fed with complete media two times per week. The colonies were counted microscopically. Cell numbers from ten different areas per plate were averaged.

**Acini assay**

The transformation ability of 67NR and 4T1 cells were studied by performing the acini assay as previously described ^1^. The cells were counted, and 5x10^3^ cells in assay medium (DMEM/F12), 2% growth factor-reduced Matrigel (Corning, catalog number 356230) basement membrane matrix, and 5 ng/ml EGF (Sigma-Aldrich, catalog number E9644) were plated on top of solidified growth factor-reduced Matrigel in each well of an 8-well chamber slide. The cells were grown in a 5% CO_2_ incubator at 37 °C. The cells were fed with assay medium containing 2% Matrigel and 5 ng/ml EGF every 4 days. The acini were counted under a microscope, and numbers from ten different areas per plate were averaged.

**Transwell migration and invasion assay**

Transmigration assays were performed on transwell filters with 67NR and 4T1 cells labeled with luciferase and RFP. To the lower compartment of the transwell filter (Costar, catalog number CLS3422-48EA), 600 µl of DMEM/F12 medium containing 0.1% FBS was added. To the upper compartment 5x10^3^ cells were added in 100 µl serum-free medium. In the stimulation assays CXCL4 (10 µg/ml) or platelets were added to the lower chamber, and the tumor cells were added to the upper chamber. For invasion assays, Matrigel invasion chambers (Corning, catalog number 354480) with 5x10^4^ cells were used. After incubation for 16 hours at 37 °C, the inserts were carefully removed, and the medium and cells within the upper compartment of the insert were gently removed with a cotton swab. The cells on the lower side of the insert membrane were fixed with 4% paraformaldehyde for 10 minutes, followed by staining with 0.1% crystal violet (Fisher Scientific, catalog number C581-25) for an additional 20 minutes. The insert was submerged in doubly distilled H_2_O for a few seconds to remove excess dye, and excess water was removed using a cotton swab. The cells on the lower side of the filter were counted under a microscope. Cell numbers from ten different areas per transwell filter were averaged.

**Isolation of CD8^+^ T cells from tumors**

Freshly harvested tumor was minced in complete media (DMEM-F12). The tissue was then digested in medium containing collagenase II (1 mg/ml, Gibco Life Technologies, catalog number 17101-015) by incubating at 37 °C for 2 hours with repeated vigorous pipetting. After the digestion, the cells were washed in 10 ml PBS with 0.5% bovine serum albumin, centrifuged at 400 x g, and placed on ice. Cell preparations were passed through a 0.44-µm sieve and were microscopically evaluated for the condition of the single-cell suspension. The cells were then stained with the Alexa Fluor 488-labeled anti-mouse CD8a (catalog number 100723) and with NucRed Dead 647 ReadyProbes Reagent (ThermoFisher Scientific) to eliminate dead cells when analyzed by FACS.

**Isolation of bone marrow cells from mice**

Bone marrow cells were isolated from femur and tibia of the BALB/c mice. The mice were anesthetized with isoflurane followed by euthanasia by cervical dislocation and an incision was made in the skin above the foot with a sterile scissors. Using sterile forceps, the skin was pulled upward and peeled off the leg. With the aid of sterile scalpel, the tissue surrounding the bone was removed carefully without damaging the bone. The leg bone was cleaned with 70% alcohol and cut in the middle. Using a sterile syringe, the bone marrow was flushed out of the bone and into a clean falcon tube using DMEM. The falcon tube was then centrifuged at 200 x g for 8 minutes at 4 °C. After centrifugation, the supernatant was discarded, and the pellet was suspended in 1 ml of erythrocyte lysis buffer for 5 minutes at room temperature to lyse red blood cells. The sample was centrifuged at 200 x g for 10 minutes at room temperature. The supernatant was discarded, and the pellet, which is devoid of red blood cells, was washed with 10 ml of PBS containing 0.1% FBS and then resuspended in 10 ml of PBS. The sample was allowed to stand undisturbed for 1 minute to allow heavy debris to settle, and the buffer containing cells was transferred to a fresh Falcon tube. The bone marrow pellet was then resuspended in complete RPMI media and counted using a hemocytometer.

**Isolation of mouse platelets**

Platelets were isolated from wildtype female BALB/c mice as previously described ^2^. Approximately 1 ml of blood was drawn from inferior vena cava into EDTA-coated vacutainers (Fisher Scientific, catalog number 02-683-99D). The blood was then centrifuged at 200 x g with no brakes applied for 20 minutes at room temperature resulting in the formation of three distinct layers: the bottom layer contained red blood cells; the middle layer contained white blood cells; and the top layer contained platelet-rich plasma. Two-thirds of the platelet-rich plasma was transferred into a new plastic tube to which HEP buffer (140 mM NaCl, 2.7 mM KCl, 3.8 mM HEPES, 5 mM EGTA, pH 7.4) at 1:1 ratio (v/v) was added. Prostaglandin E1 (Sigma, catalog number P5515-1MG) was added at a final concentration of 1 µM to prevent platelet activation. The tube was then mixed very gently by inverting and centrifuged at 100 x g for 15-20 minutes at room temperature. The supernatant was transferred into a new plastic tube and centrifuged at 800 x g for 15-20 minutes at room temperature with no brakes applied. The resulting platelet pellet was rinsed with platelet wash buffer (10 mM Na_3_C_6_H_5_O_7_, 150 mM NaCl, 1 mM EDTA, 1% (w/v) glucose, pH 7.4) and carefully resuspended in Tyrode’s buffer (134 mM NaCl, 12 mM NaHCO_3_, 2.9 mM KCl, 0.34 mM Na_2_HPO_4_, 1 mM MgCl_2_, 10 mM HEPES, pH 7.4) containing 5 mM glucose, and 3 mg/ml bovine serum albumin. The platelets were counted using a hemocytometer.

**CXCR3 inhibition**

AMG487 was used to inhibit CXCR3 function. AMG was prepared in 20% hydroxypropyl-b-cyclodextrin in water and was administered at 5 mg/kg i.p. to BALB/c wild type mice with palpable 4T1 tumors twice a day on days 13–17, followed by daily dosage until the termination of the experiment. 20% hydroxypropyl-b-cyclodextrin in water was used as a control.

**SUPPLEMENTARY REFERENCES**

1 Debnath, J., Muthuswamy, S. K. & Brugge, J. S. Morphogenesis and oncogenesis of MCF-10A mammary epithelial acini grown in three-dimensional basement membrane cultures. *Methods* **30**, 256-268, (2003).

2 Haemmerle, M., Taylor, M. L., Gutschner, T., Pradeep, S., Cho, M. S., Sheng, J. *et al.* Platelets reduce anoikis and promote metastasis by activating YAP1 signaling. *Nat Commun* **8**, 310, (2017).
